# Comparative Cytogenetic of the 36-Chromosomes Genera of *Orchidinae* Subtribe (Orchidaceae) in the Mediterranean Region: A Summary and New Data

**DOI:** 10.3390/plants12152798

**Published:** 2023-07-28

**Authors:** Alessio Turco, Antonella Albano, Pietro Medagli, Robert Philipp Wagensommer, Saverio D’Emerico

**Affiliations:** 1 Department of Biological and Environmental Sciences and Technologies, University of the Salento, 73100 Lecce, Italy; alessio.turco@unisalento.it (A.T.); antonella.albano@unisalento.it (A.A.); pietro.medagli@unisalento.it (P.M.); 2 Faculty of Education, Free University of Bozen-Bolzano, 39042 Brixen-Bressanone, Italy; 3 Aldo Moro University of Bari, 70125 Bari, Italy; sdeme@yahoo.it

**Keywords:** *Anacamptis*, Fluorescent in situ hybridization, *Himantoglossum*, Karyosystematic, *Ophrys*, *Serapias*

## Abstract

This article provides a summary of the current knowledge on the cytogenetics of four genera, which are all composed of 36 chromosomes, within the Orchidinae subtribe (Orchidaceae). Previous classical studies have revealed differences in karyomorphology among these genera, indicating genomic diversity. The current study includes an analysis of the current knowledge with an update of the karyotype of 47 species with 36 chromosomes from the genera *Anacamptis*, *Serapias*, *Himantoglossum*, and *Ophrys.* The study discusses comparisons of karyotypes among these genera that used traditional techniques as well as karyotype asymmetry relationships with various asymmetry indices. Additionally, the study reports new findings on polyploidy in *Anacamptis pyramidalis* and *Serapias lingua*, which were observed through karyotype and meiotic metaphase analyses in EMC. Moreover, the study detected B chromosomes for the first time in *A. papilionacea* and *A. palustris*. The article also describes the use of fluorescent in situ hybridization in some specimens of *A. papilionacea* and *A. collina* to locate different sites of the 18S-5.8S-25S rDNA and 5S rDNA ribosomal complexes on chromosomes. The information derived from these cytogenetic analyses was used to refine the classification of these orchids and identify evolutionary relationships among different species and genera.

## 1. Introduction

The Orchidaceae Juss. family is composed of over 28,000 plant species belonging to 763 genera and is widely distributed throughout the world, with a significant concentration in the humid tropics [1]. Orchidaceae is classified under the order Asparagales Link, a group of monocotyledonous flowering plants that also encompasses, e.g., the Asparagaceae Juss. and Iridaceae Juss. families. While morphology was previously the main means for describing new species, nowadays molecular analyses (DNA sequences) are more and more frequently included in new species descriptions [2,3]. Nevertheless, descriptions of new orchid taxa should include both genetic and morphological studies.

The *Orchidinae* Verm. subtribe consists of approximately 53 genera and over 1800 species [1,4], making it one of the most species-rich subtribes within the Orchidaceae family. These orchids are predominantly terrestrial, growing in a variety of habitats, including grasslands, forests, and even alpine regions. Although *Orchidinae* species are primarily found in temperate and Mediterranean regions of Europe, Asia, and Africa, some species have also been discovered in the Americas and Australia [5,6].

One of the most distinctive characteristics of *Orchidinae* orchids is their highly specialised pollination mechanisms [7]. Indeed, many species within this subtribe have evolved remarkable adaptations for attracting and manipulating their pollinators, often involving intricate flower morphology and then complex mimicry attraction systems, in some cases highly specialised [8,9]. In this context, indeed, some *Orchidinae* genera show pollinator attraction systems at different levels of specialization, such as those belonging to the genus *Ophrys* L., which are known for their remarkable ability to mimic the appearance and scent of female insects to attract male pollinators [10,11,12].

The taxonomy of *Orchidinae* has been a subject of ongoing research and debate among botanists as molecular phylogenetic studies continue to reveal new insights into the relationships among its genera and species [3,13]. As a result, the circumscription and classification of this subtribe have undergone significant changes in recent years, with some genera being redefined [14].

This subtribe is present in Europe and the Mediterranean Basin, with genera such as *Anacamptis* Rich., *Chamorchis* Rich., *Dactylorhiza* Neck. ex Nevski, *Gennaria* Parl., *Gymnadenia* R.Br. in W.T.Aiton (including *Nigritella* Rich.), *Herminium* L., *Himantoglossum* Spreng. (including *Comperia* Koch and *Barlia* Parl.), *Neotinea* Rchb.f., *Ophrys* L., *Orchis* L. s.s., *Platanthera* Rich., *Pseudorchis* Ség., *Serapias* L., and *Traunsteinera* Rchb. [15].

Cytogenetic studies on *Orchidinae* genera have been limited, with only 13 of the 17 extant genera having known chromosome numbers and karyotypes, despite their importance for morphological characters. The *Orchidinae* subtribe is interesting for investigating karyotype evolution due to its global distribution of species, different basic chromosome numbers, and various ploidy levels [16,17,18]. In fact, chromosome number variation in *Orchidinae* is notable, as many genera have high ploidy levels and variable base numbers. The known basic numbers in *Orchidinae* are x = 14, 16, 18, 20, and 21, with polyploid series ranging from 2n = 54 to 168 chromosomes [16].

Comparisons of the karyological characteristics of different species of Orchidaceae can reveal interesting evolutionary hypotheses in some groups of species. For example, pairs of species joined by a common pollinator have divergent karyotypes (e.g., *Anacamptis palustris* (Jacq.) R.M.Bateman, Pridgeon, and M.W.Chase/*A. laxiflora* (Lam.) R.M.Bateman, Pridgeon, and M.W.Chase), while pairs of species that attract different pollinators have more similar karyotypes (e.g., *Ophrys fusca* s.l./*O. tenthredinifera* s.l.) [19].

Differential banding techniques have also revealed variations in the constitutive heterochromatin distribution in the chromosomal sets of many plant groups [20] and these variations have been useful in obtaining phylogenetic correlations [21]. In DNA, heterochromatic regions consist mostly of repeating base sequences, and quantitative variations in the genome can indicate karyotype mutations [22]. Cytogenetic analysis has been especially useful in phylogeny studies in orchids, greatly influencing the taxonomy and classification of the *Orchidinae* subtribe [4,13,23].

This work reviews all available cytogenetic data for the 36-chromosome genera of the subtribe *Orchidinae* and combines previous findings with later and updated data to interpret chromosome evolution and speciation.

## 2. Results

**Figure 1:**
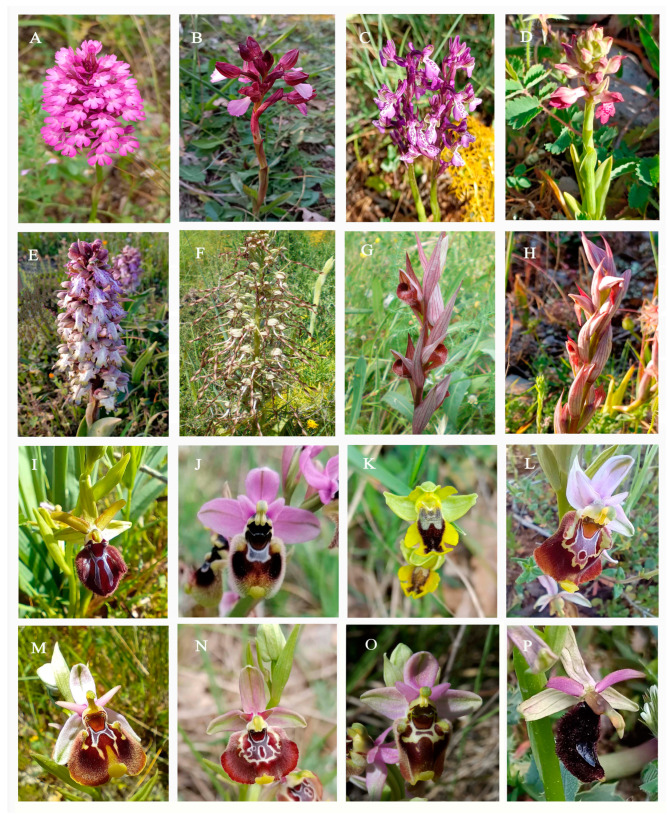
(**A**) *Anacamptis pyramidalis*; (**B**) *A. papilionacea*; (**C**) *A. morio*; (**D**) *A. coriophora*; (**E**) *Himantoglossum robertianum*; (**F**) *H. hircinum*; (**G**) *Serapias bergonii*; (**H**) *S. parviflora*; (**I**) *Ophrys incubacea*; (**J**) *O. tenthredinifera*; (**K**) *O. sicula*; (**L**) *O. apulica*; (**M**) *O. peucetiae*; (**N**) *O. celiensis*; (**O**) *O. apulica* × *O. tenthredinifera*; (**P**) *O. bertolonii*.

In Figure 1, several species from the genera *Anacamptis*, *Himantoglossum*, *Serapias*, and *Ophrys* are depicted.

Table 1 and Table 2 present all the analysed taxa, highlighting the karyotype and related parameters of 47 species. Some of these species provide new karyological data, while for others the previous data were updated and recalculated using the IdeoKar program. In the *Ophrys* group, the parameters of some species have been taken from Deniz et al. [24].

Methods used in this approach include the Feulgen stain for chromosomal counting and karyomorphological analysis, Giemsa band staining to detect constitutive heterochromatin, DAPI and CMA3 fluorochrome staining to identify A-T and G-C rich regions with repeated sequences, and in situ hybridization to observe ribosomal sites such as 18S-5.8-25S rDNA and 5S rDNA.

### 2.1. Anacamptis s.l.

**Figure 2:**
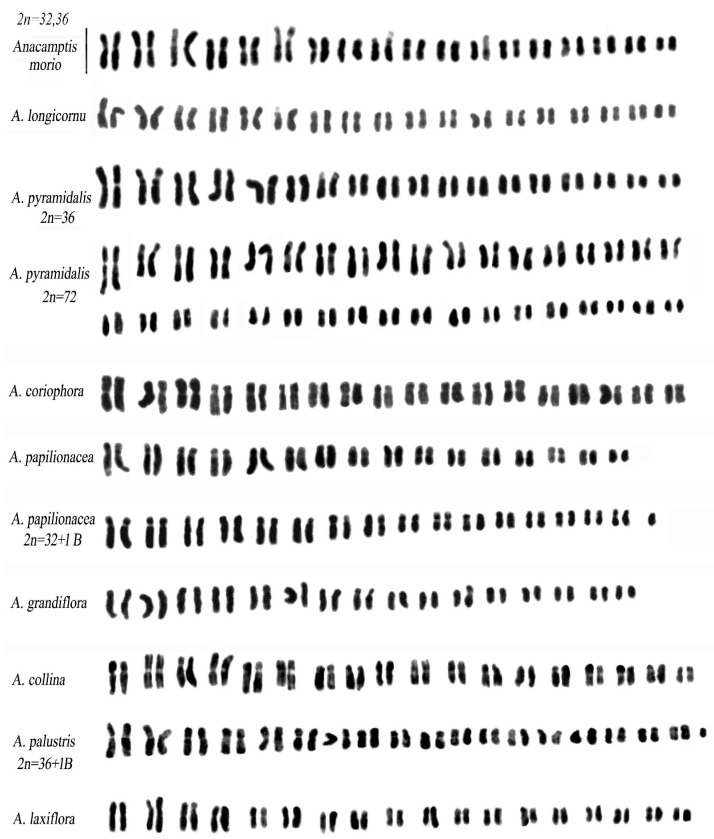
Karyotypes in *Anacamptis* species. Scale bar = 5 µm.

A karyomorphological study of *Anacamptis* species confirmed a standard karyotype with variations in the number of submetacentric chromosomes (Figure 2). *A. collina* and *A. coriophora* showed similarity in the first two chromosome pairs, with a secondary constriction on the long arm and an equal number of submetacentrics. *A. pyramidalis* differed from these species by having a secondary constriction on the short arm of the second pair and many metacentric chromosomes similar to those of *A. laxiflora*, *A. longicornu* (Poir.), R.M.Bateman, Pridgeon, and M.W.Chase, *A. morio*, and *A. palustris*. *A. longicornu* and *A. morio* had very similar karyology, with no secondary constrictions in the first pairs. *A. laxiflora* and *A. palustris* had similar karyotypes with a secondary constriction on the second pair, similar to *A. pyramidalis*. *A. papilionacea* had a substantially different karyotype from all other *Anacamptis* species, with a secondary constriction on the long arm of the first pair and a constriction on the short arm. The karyotype had many submetacentric pairs of chromosomes and one subtelocentric pair.

**Figure 3:**
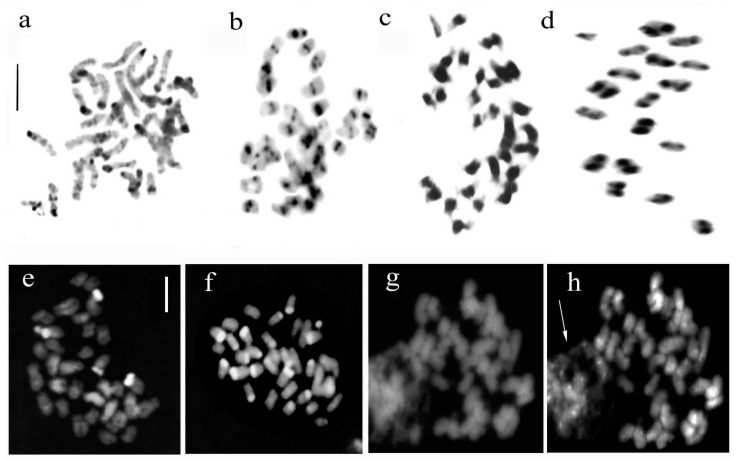
(**a**–**d**). Banding technique with Giemsa. (**a**) *Anacamptis coriophora*; (**b**) *Ophrys tenthredinifera*; (**c**,**d**) *Serapias vomeracea*, in (**d**) metaphase I of meiosis with 18 bivalents, to note constitutive heterochromatin blocks; (**e**–**g**). Banding with fluorochrome DAPI. (**e**) *Anacamptis papilionacea*; (**f**) *A. coriophora*; (**g**) *Ophrys sicula*. Fluorochrome CMA3. (**h**) *O. sicula*; the arrow indicates an interphase nucleus containing numerous chromocentres. Scale bar = 5 µm.

The karyomorphological similarities in *A. laxiflora*, *A. longicornu*, *A. morio*, *A. palustris*, and *A. pyramidalis* were confirmed through differential staining methods. These species had a low content of constitutive heterochromatin, indicating their symmetrical karyotypes with a prevalence of metacentric chromosomes. In contrast, *A. coriophora* and *A. papilionacea* exhibited asymmetrical karyotypes with a prevalence of submetacentric chromosomes and a greater amount of heterochromatic content (Figure 3a,e,f). These two species showed positive reactions to DAPI banding, indicating the presence of heterochromatin rich in Adenine-Thymine (A-T) bases. Giemsa and fluorochrome staining methods revealed considerable heterochromatin content in *A. coriophora*, *A. fragrans* (Pollini), R.M.Bateman, and *A. papilionacea*. Heterochromatic bands were seen in three pairs of chromosomes in *A. papilionacea* and predominantly at the telomeric position in *A. coriophora.* These findings suggest different evolutionary processes within the genus *Anacamptis* despite the similarity found between the chromosomal complements, with variations in the morphology of chromosomes and the content of heterochromatin.

In *A. pyramidalis*, an interesting case of polyploidy has been observed, with diploids having 2n = 2x = 36 chromosomes and polyploid individuals having 2n = 3x = 54 and 2n = 4x = 72 chromosomes.

Further analysis of specimens of *A. pyramidalis* (2n = 72) reported 36 bivalents in metaphase I, with an arrangement of the chromosomes in pairs in the karyotype rather than four. Therefore, considering the latest data, classical cytogenetic studies tend to suggest a present allotetraploidy in the 72-chromosome cytotype of *Anacamptis pyramidalis* (Figure 2).

Additionally, a specimen of *A. papilionacea* with chromosome number 2n = 32 + 1B and a specimen of *A. palustris* with an accessory chromosome (2n = 36 + 1B) were found, which are the first cases reported in the *Anacamptis* group (Figure 2).

### 2.2. Serapias L.

**Figure 4:**
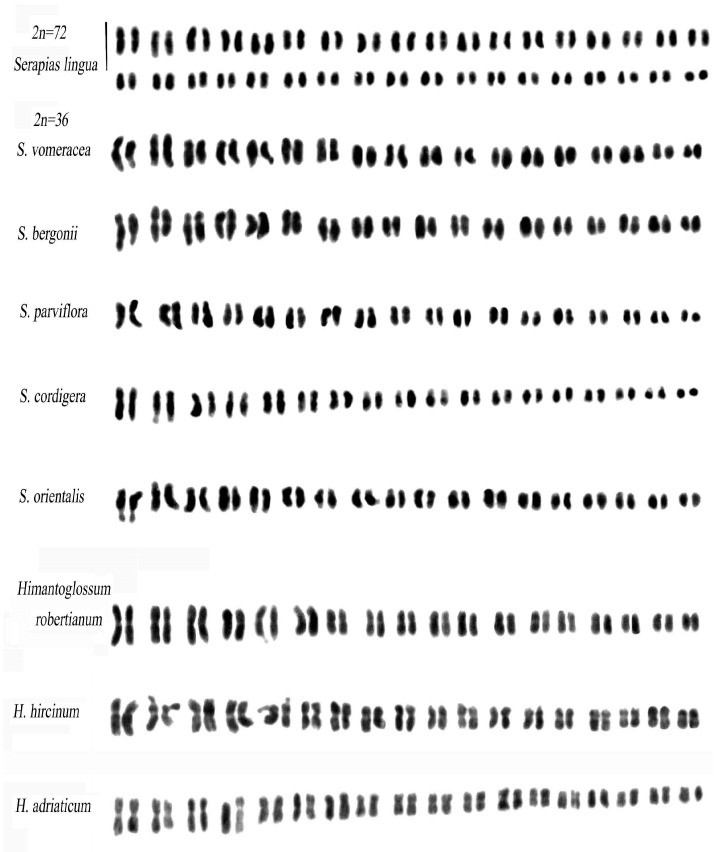
Karyotypes of *Serapias* and *Himantoglossum* species. Scale bar = 5 µm.

In the *Serapias* group, metaphase I chromosome plates were successfully obtained only in *S. lingua*, specifically for the polyploid species. Interestingly, these plates exhibited 36 bivalents during meiosis, suggesting a potential occurrence of allopolyploidization. The meiotic data were further supported by karyotype constructions, where chromosomes were paired instead of grouped in fours (Figure 4).

The Feulgen method was used to analyse the karyomorphology of the species, and it revealed a complex karyotype with moderately asymmetrical chromosomes. The karyotype is mainly composed of submetacentric chromosomes, with several pairs of chromosomes having secondary constrictions on the long arm and one pair having secondary constrictions on both the short and long arms. Among species with 36 chromosomes, *S. bergonii*, *S. orientalis* s.l., and *S. vomeracea* have fewer metacentric chromosomes compared to *S. parviflora* and *S. cordigera*, which have a higher number of metacentrics.

The Giemsa C-banding method was used to observe the distribution of constitutive heterochromatin and to show the presumed secondary constrictions. The C-banding results in *S. bergonii*, *S. cordigera*, *S*. x *intermedia* subsp. *hyblaea*, *S. lingua*, *S. orientalis* s.l., *S. parviflora*, *S. politisii*, and *S. vomeracea* showed broad centromeric bands in most of the chromosomes (Figure 3c,d).

### 2.3. Himantoglossum s.l. (Including Comperia and Barlia)

Within the *Himantoglossum* group, the karyotypes of the three species studied exhibit striking similarities, characterized by predominantly metacentric chromosomes. These findings strongly imply a closely related phylogenetic association among them.

Interestingly, all analysed species have symmetric karyotypes and exhibit heterochromatic properties of *Anacamptis* s.l.

In this group of species, the Giemsa banding method shows poor heterochromatic content. *H. hircinum* has a slightly asymmetric karyotype and modest constitutive heterochromatin. Comparisons with *H. robertianum* reveal similar karyomorphologies, but the latter species has a more symmetrical karyotype (Figure 4).

### 2.4. Ophrys L.

**Figure 5:**
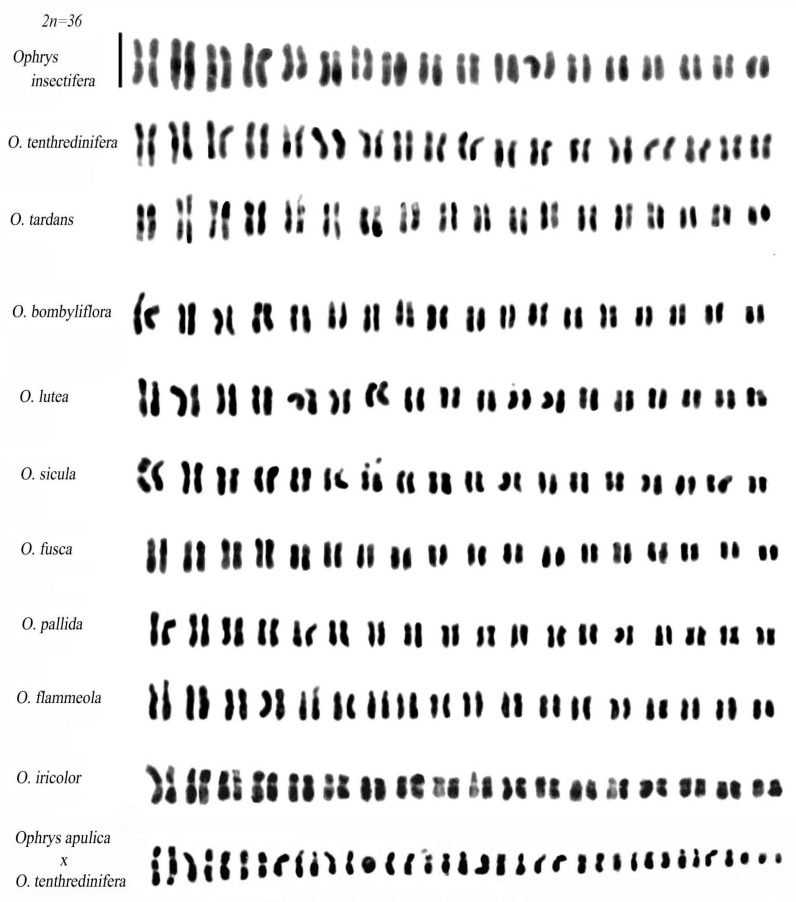
Karyotypes of *Ophrys* species. Scale bar = 5 µm.

**Figure 6:**
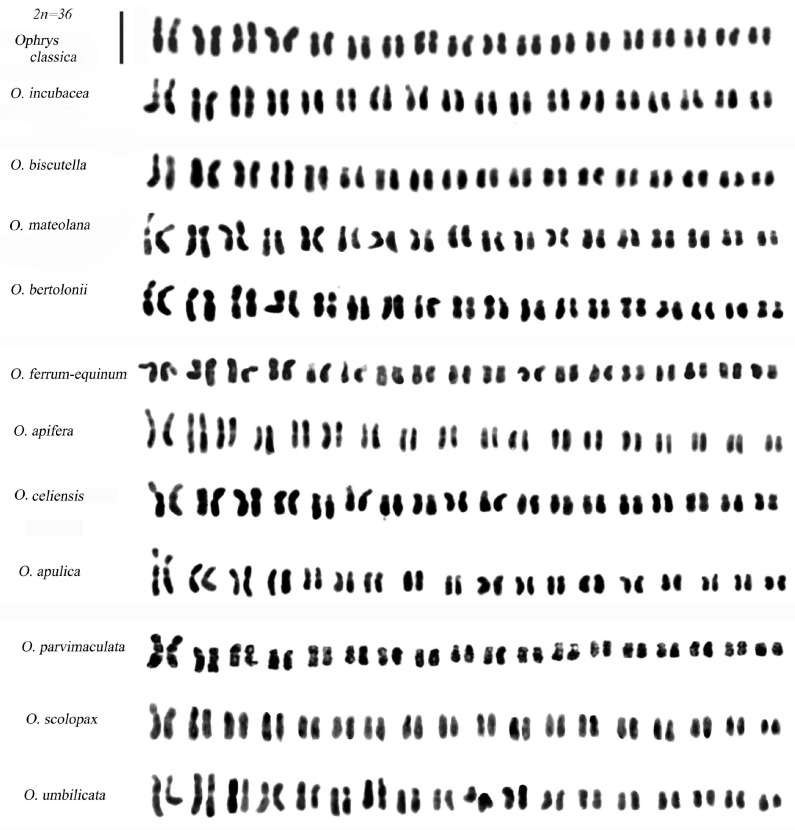
Karyotypes of further *Ophrys* species. Scale bar = 5 µm.

Most species within the *Ophrys* genus are diploid, with a base chromosomal number of x = 18 resulting in a diploid number of 2n = 36 (Figure 5 and Figure 6).

Typically, the standard karyotype of *Ophrys* is characterised by three pairs of chromosomes with secondary constriction. The first pair is typically a metacentric or submetacentric chromosome with an evident constriction on the short arm (shown in Figure 5 and Figure 6). However, there is notable variation in the morphology of the first pair of chromosomes within the *Ophrys* group, including differences in the size of the secondary constriction. For instance, the first pair in the sections *Apiferae*, *Araniferae*, and *Fuciflorae* is distinguishable from other *Ophrys* groups due to the larger size of the secondary constriction, whereas the *O. fusca*-*O. lutea*-*O. omegaifera* complex has a medium secondary constriction on the short arm of its first pair [25]. The first pair in *O. tenthredinifera* is characterized by a clear secondary constriction on the long arm ([26] and this work), which is of great importance in recognizing the chromosomes of interspecific hybrids derived from the cross between *O. tenthredinifera* and other *Ophrys* species (e.g., in the hybrid *O. apulica* and *O. tenthredinifera*) (Figure 5). Notably, the first pair of *O. tardans* is similar to that of *O. tenthredinifera*, consisting of a secondary constriction on the long arm. However, unlike other *Ophrys* species examined, no secondary constrictions were observed in the first pair of chromosomes in *O. insectifera*.

The Giemsa banding technique was applied to the analysed *Ophrys* species, which revealed centromeric bands in all chromosomes. Some taxa also exhibited terminal and subterminal bands (as shown in Figure 3b). Moreover, the distribution of constitutive heterochromatin differed significantly among the species belonging to different sections of the genus. While the DAPI fluorochrome did not yield any results in terms of heterochromatin content (Figure 3g), the chromomycin (CMA3) was effective in highlighting sites rich in G-C bases, including centromeric and intercalary bands (as shown in Figure 3h). Silver staining was employed to identify active nucleolar organising regions (NORs) in the chromosomes. The number of NORs in chromosome complements provides valuable insights into the genome composition of various plants [27]. This technique revealed a minimum of two nucleoli in *Ophrys apifera* and *O. bombyliflora*, three nucleoli in *O. tenthredinifera*, and four nucleoli in *O. fusca*, *O. lutea*, and *O. praecox* ([25], this work).

### 2.5. Diagram of the Morphometric Parameters A1 (Intrachromosomal Index) and A2 (Interchromosomal Index); Mca (Mean Centromeric Asymmetry) and CVcl (Coefficient of Variation of Chromosome Length).

**Figure 7:**
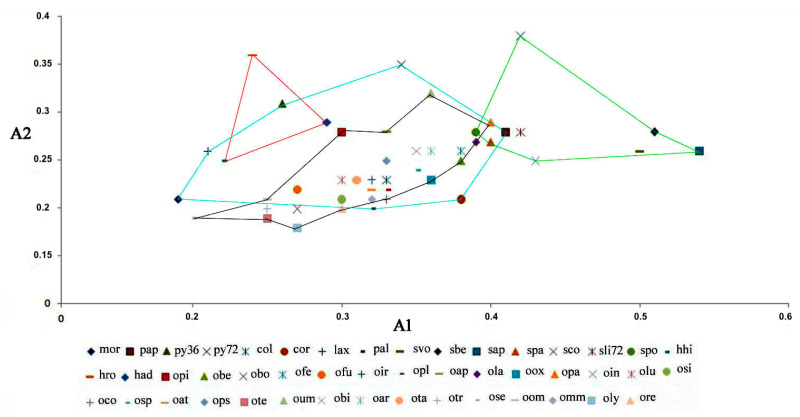
Diagram of the A1 and A2 values of the karyotypes of the species examined. Specifically, the red line represents *Himantoglossum*, the blue line *Anacamptis*, the black line *Ophrys*, and the green line *Serapias* taxa (for the meaning of the codes, see Table 1 and Table 2).

**Figure 8:**
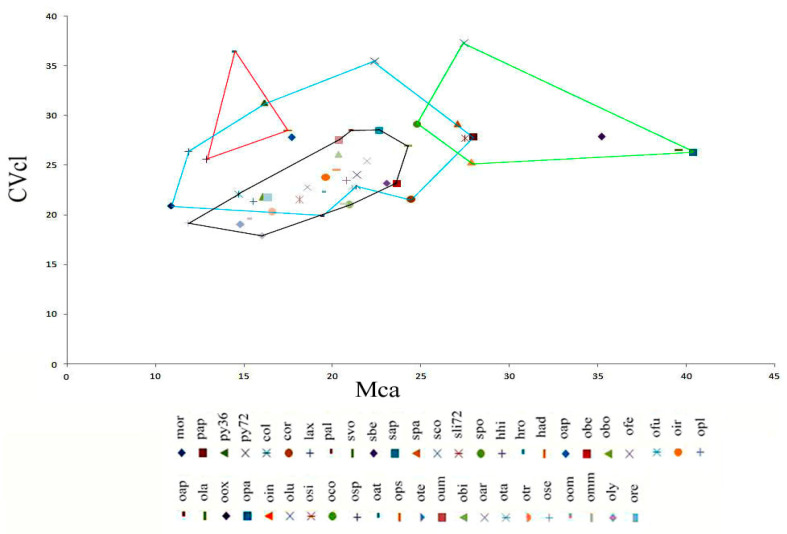
Diagram of the Mca and CVcl values of the karyotypes of the species examined. Specifically, the red line represents *Himantoglossum*, the blue line *Anacamptis*, the black line *Ophrys*, and the green line *Serapias* taxa (for the meaning of the codes, see Table 1 and Table 2).

We used the asymmetry indices A1, A2, Mca, and CVcl to construct the diagrams (Figure 7 and Figure 8). The diagram depicted in Figure 7 and Figure 8 highlights the four genera, each represented by a distinct coloured line. The two diagrams exhibit striking similarity when it comes to the *Anacamptis*, *Himantoglossum*, and *Serapias* groups, with a minor variation observed for the *Ophrys* group.

Upon analysing the diagrams in Figure 7 and Figure 8, it becomes evident that *Anacamptis*, *Himantoglossum,* and *Ophrys* share a similar pattern of asymmetry indices. However, *Ophrys* shows a higher degree of homogeneity than the *Anacamptis* group, which shows significant discontinuity. In particular, the *Serapias* group has a higher A1 and Mca asymmetry index, making it interesting and noteworthy.

### 2.6. Fluorescence In Situ Hybridization (FISH) in Some Species

**Figure 9:**
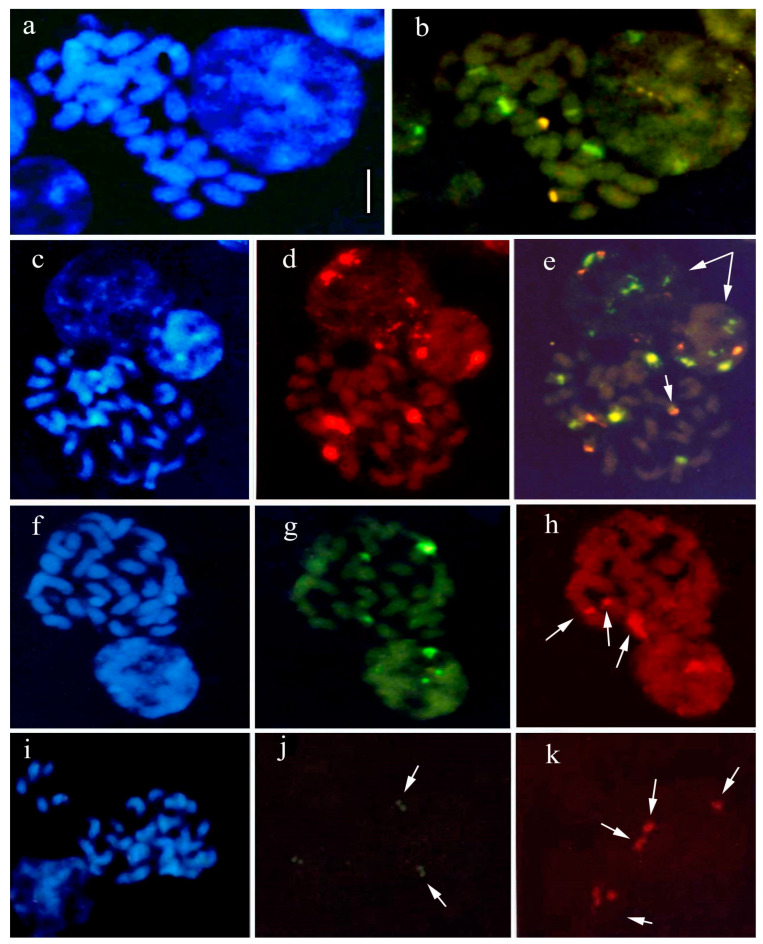
In situ hybridization is applied to the chromosomes of orchid species. Blue DAPI staining shows chromosomal DNA (**a**,**c**,**f**,**i**), respectively, in *Anacamptis papilionacea* (**a**,**c**), *A.* × *gennarii* (**f**), and *A. collina* (**i**). Red and green signals show sites of hybridization of 18S-25S rDNA and 5S rDNA, respectively (**b**,**d**,**e**,**g**,**h**,**j**,**k**): *A. papilionacea* (**b**) two 18S-25S rDNA sites and four 5S rDNA sites; *A. papilionacea* (**d**,**e**) three 18S-25S rDNA sites and five 5S rDNA sites; (**e**) long arrows indicate interphase nuclei sites; and a short arrow indicates one 5S rDNA site adjacent to the 18S-25S rDNA site; *A*. × *gennarii* (**g**,**h**) three 18S-25S rDNA sites; and three 5S rDNA sites, (**h**) arrows indicate 18S-25S rDNA sites; *A. collina* (**j**,**k**) three 18S-25S rDNA sites and two 5S rDNA sites, long arrows indicate 5S rDNA and 18S-25S rDNA sites; respectively, (**k**) short arrow indicate interphase nucleus sites. Scale bar = 5 µm.

The method of detecting recurring DNA sequences was applied to some *Anacamptis* species, including the hybrid *A*. × *gennarii* and its parents *A*. *morio* and *A. papilionacea*, which confirmed previous karyological studies (Figure 9).

Different 5S rDNA and 18S-5.8S-25S rDNA sites were also observed in *A. papilionacea*. The study of this species, the only *Anacamptis* species with 2n = 32 chromosomes, using pTa71 and pTa794 probes, produced interesting results. Other populations of this species were examined, and the previous findings of four/five 5S rDNA sites and two 18S-25S sites were confirmed. Additional 5S rDNA and 18S-5.8S-25S rDNA sites were also observed in some samples, with a weakly stained 5S rDNA site adjacent to the 18S-5.8S-25S rDNA site (Figure 9d,e). In interphase nuclei, the signals were distributed all over the nucleus (Figure 9a–e). One major and one minor 5S rDNA site were identified in all tested samples. In addition, previous analyses of the hybrid *A*. × *gennarii* were confirmed (Figure 9f–h).

Similar results were also observed in specimens of *A. collina* at two different stations. Indeed, recent analyses showed an additional 18S-5.8S-25S rDNA site (Figure 9i–k) compared to previous findings, which only identified two sites.

## 3. Discussion

In recent years, significant progress has been made in understanding the phylogeny of various plant groups using molecular cytogenetics, particularly fluorescent in situ hybridization, as evidenced by studies [28,29]. Conventional methods such as the Feulgen technique and Giemsa banding have also provided valuable parameters for comparing the karyotypes of species and solving taxonomic problems in certain genera of the subtribe *Orchidinae*.

The classification of the Orchidaceae family has undergone multiple revisions using phylogenetic analyses of ecological and morphological characters. To further refine the classification at the genus or species level, a multidisciplinary approach, including classical and molecular cytogenetics, can be utilized.

Previous studies on the *Orchis* s.l. group using conventional cytogenetic analysis have shown that the genus has three basic chromosomal numbers: x = 16, 18, and 21. Additionally, the species vary in chromosome size and morphology. Species with 2n = 36 have slightly larger chromosomes with an evident centromere compared to those with 2n = 42, which are characterized by complex karyomorphology [30]. Further studies using nucleotide sequence analysis have confirmed these cytological findings and divided *Orchis* s.l. into three taxonomic groups: *Anacamptis* s.l. (2n = 32, 36); *Neotinea* s.l.; and *Orchis* s.s. (42 chromosomes) [13,31,32].

Due to the good morphology of the chromosomes, the species of the *Anacamptis* group have been the most studied from a karyological point of view [30,33,34]. Indeed, numerous staining methods have been applied to these species, ranging from the traditional ones such as the Feulgen technique and Giemsa banding to techniques using the fluorochromes DAPI, CMA3, and FISH [34,35,36].

The *Anacamptis* s.l. group displays distinctive evolutionary patterns in the development of taxonomic entities. Searches have been helpful in identifying unique characteristics within the same group by studying the morphology of chromosomes and the distribution of heterochromatin. Notably, species with asymmetrical karyotypes, such as *A. coriophora* and *A. papilionacea* (Figure 2a,e,f), exhibit a greater amount of heterochromatin than species with symmetrical karyotypes like *A. laxiflora*, *A. longicornu*, and *A. morio*. Despite the differences, Bateman’s observation of the karyomorphological diversity within the Anacamptis group is accurate, as the basic structure remains similar [36].

Only two interspecific hybrids in Anacamptis s.l., namely *Anacamptis* × *gennarii* (Rchb. f.) H. Kretzschmar, Eccarius, and H. Dietr. (*A. morio* × *A. papilionacea*) and *Anacamptis* × *murgiana* Medagli, D’Emerico, Ruggiero, and Bianco (*A. collina* × *A. morio*), have been subjected to karyological analysis.

*Anacamptis* × *gennarii*, with 2n = 34, is a commonly occurring hybrid in populations where the parental species *Anacamptis morio* and *A. papilionacea*, which have a chromosomal number of 2n = 36 and 2n = 32, respectively, are sympatric. Meiotic analysis of M-I revealed numerous univalents and few bivalents, indicating reduced homology between parental genomes. There were no intermediate counts in the analysed specimens, suggesting that the hybrid did not interbreed with its parents. The hybrid specimens displayed an extremely variable complement, indicating substantial differences from the presumed parental species and resulting in the sterility of the hybrid specimens. These differences probably play a crucial role in preventing introgression. The hybridization process in *A*. × *gennarii* was particularly noteworthy regarding phenotype. Some specimens exhibited characteristics of a single parent species, while others displayed intermediate or entirely new characters [37,38]. Another interesting discovery was the analysis of an allotriploid of *A.* × *gennarii*, which exhibited 2n = 52 chromosomes in somatic metaphases and 18 bivalent and 16 univalent chromosomes in Metaphase I of meiosis [39].

In addition to *A. × gennarii*, there was another case of interspecific hybridization observed through traditional karyological analyses. Hybridization also occurred between *A. collina* and *A. morio* (*A. × murgiana*), with both parents having 2n = 36 chromomsomes. Some hybrid specimens showed intermediate morphological characters between the presumed parental species, with one specimen having an allotriploid chromosomal number of 2n = 3x = 54 and the remaining specimens having a diploid number of 2n = 36. The karyotype of the triploid hybrid specimen had 36 typical chromosomes from *A. collina* and 18 chromosomes from *A. morio* [37].

Furthermore, during several analyses of orchid populations, autotriploid specimens were observed in the species *A. coriophora*, *A. laxiflora*, and *A. pyramidalis*. These triploid specimens had a chromosomal number of 2n = 3x = 54 and were characterised by trivalent formations in Metaphase I in EMC. [33,37].

A new species, *Anacamptis berica* Doro, with a chromosomal count of 2n = 4x = 72, has recently been discovered in this genus [40]. However, there is currently no information available regarding the meiotic counts of this species. Meiosis is important in cytogenetics; indeed, meiotic configurations can provide information in chromosome research through the construction of cytogenetic maps or determine the relationship between the chromosomes of related species [41].

The aforementioned karyomorphological structure is also observed in the *Himantoglossum* group, highlighting a phylogenetic proximity to *Anacamptis s.l.*, at least in terms of the basic karyotype. *Himantoglossum* s.l. is a group of species [13,42] that has a chromosomal number of 2n = 36, according to studies conducted by D’Emerico et al. [43]. However, Ströhlein and Sundermann [44] reported a chromosomal number of 2n = 30 in *H. comperianum* (Steven) P.Delforge. The Kew Plants of the World Flora Online [45] recognises seven species of this genus in the Mediterranean region, but only three species (*H. adriaticum*, *H. hircinum*, and *H. robertianum*) are known to have the chromosomal number 2n = 36. Aneuploidy with 2n = 36 + 1B has been reported in *H. adriaticum* and *H. hircinum* [37,46]

In contrast, the genera *Ophrys* and *Serapias* both have 2n = 36 chromosomes and exhibit relatively uniform karyomorphology.

*Serapias* is a group of Orchidaceae that is mainly found in the Mediterranean basin, the Canaries, and the Azores [47]. It is a taxonomically challenging group for infragenus identification that has also undergone numerous taxonomic revisions in recent years, which have led to the description of new species [48]. In this genus, most species have a diploid chromosomal number of 2n = 36, with the exception of some species such as *S. gregaria*, *S. lingua*, *S. olbia* Verguin, and *S. strictiflora* Weilwitsch ex Da Veiga that have a polyploid number of 2n = 4x = 72 [49,50,51,52], while *S*. × *intermedia* subsp. *hyblaea* Cristaudo, Galesi, and R.Lorenz, and *S.* × *todaroi Tineo* (*S. lingua* × *S*. *parviflora*) have a triploid complement of 2n = 3x = 54 [52,53]. Metaphase I chromosome plates were obtained only in *S*. *lingua* for polyploid species, which showed 36 bivalents during meiosis, indicating a possible allopolyploidization.

A new species called *S. ausoniae* Gennaio & Pellegrino has been recently described [54], which is morphologically similar to *S. parviflora* and has a chromosome number of 2n = 4x = 72. Molecular analysis suggests that this species has an autopolyploid origin. However, in contrast to *S. lingua*, no EMC meiotic plates were obtained [54].

Giemsa banding in all *Serapias* species revealed broad centromeric bands in most complement chromosomes, indicating that the species form a homogeneous group. This karyomorphology is most likely a result of chromosomal rearrangements. Interestingly, in the genus *Serapias*, the amount of constitutive heterochromatin can be correlated with the asymmetric karyotype. The presence of large centromeric heterochromatic bands, along with an asymmetric karyotype, indicates recent structural rearrangements [44,52], which are supported by molecular analyses [55].

The genus *Ophrys*, on the other hand, displays a moderately asymmetrical karyotype and constitutive heterochromatin in the centromeric position.

Based on the data available for the first pair of chromosomes of the genus *Ophrys*, it is possible to distinguish three standard karyotypes, each with a different secondary constriction. These distinct features can be used to identify the three groups within the genus (e.g., *O. fusca*-*O. lutea*-*O. omegaifera* complex, *Tenthrediniferae* section, and *Apiferae*, *Araniferae*, and *Fuciflorae* sections). The karyotypes of studied taxa within the *Ophrys* genus show a gradual progression from symmetrical to asymmetrical, with a higher number of metacentric chromosomes in the *O. fusca*-*O. lutea-O. omegaifera* group, *O. bombyliflora*, and *O. tenthredinifera*. Conversely, *O. bertolonii* and *O. insectifera* exhibit a higher number of submetacentric chromosomes. Additionally, species within the *O. fusca*-*O. lutea*-*O. omegaifera* complex, belonging to the subgenus *Ophrys*, possess a chromosome with a modest secondary constriction on the short arm as part of their first pair [25].

Studies have shown that the species in the *O. fusca*-*O. lutea*-*O. omegaifera* group have the most symmetrical karyotype, in contrast to other sections of the genus *Ophrys* that exhibit normal progressive asymmetry of the karyotype. Recent cytogenetic analysis suggests that, following multiple evolutionary factors, the karyotype of the species in the Pseudophrys section has likely differentiated from an asymmetric to a symmetrical karyotype, unlike the norm that tends towards asymmetrical from symmetrical forms [56,57,58].

There have been reported cases of polyploidy in the *O. fusca*-*O. lutea*-*O. omegaifera* complex, where some French and Iberian populations have a chromosome number of 2n = 4x = 72 or 2n = 5x = 90 [50]. In contrast, Italian populations of the *Ophrys* genus have been found to be diploid, apart from *O. lupercalis* Devillers-Terchuren and Devillers, which has a chromosome number of 2n = 72 in the Gargano Promontory [58]. Additionally, a specimen of *Ophrys tenthredinifera* exhibited an autotriploid number of 2n = 3x = 54, while cases of somatic aneuploidy with chromosome numbers of 2n = 37, 38, or 39 have also been documented [58]. Various studies have examined around 50 species of the *Ophrys* genus, including research conducted by Bianco et al. [26,59,60], Bernardos et al. [50], D’Emerico et al. [25], Deniz et al. [24], Greilhuber & Ehrendorfer [61], and Turco et al. [62].

The *Ophrys* species that were analyzed exhibited Giemsa banding patterns, which comprised constitutive heterochromatin located at the centromeric and subtelomeric regions. Additionally, fluorochrome staining revealed that only CMA3 displayed both centromeric and subtelomeric heterochromatic segments. Some researchers have suggested that the G-C content of chromosomes may have ecological relevance and could have played a significant role in the evolution of Earth’s biota [63].

The available data on the basic karyotype of the *Anacamptis*, *Ophrys*, and *Serapias genera* indicate a more differentiated karyotype in the *Ophrys* genus. This differentiation could be one of the reasons why there are no intergeneric hybrids between entities of the *Ophrys* genus and entities of other genera. If chromosomal rearrangements play a significant role in karyotype evolution, the presented cytogenetic data suggests that the *Anacamptis* s.l. group is a relatively ancestral group of entities compared to the *Ophrys* group and has not yet completed its evolution.

Regarding *Himantoglossum hircinum* and *H. robertianum*, their asymmetry indices, karyological formulas, modest constitutive heterochromatin, and few discriminating data visible only with silver staining and FISH are so similar that it is difficult to distinguish between them clearly [35].

The technique employed for detecting recurring DNA sequences is a significant approach for examining specific repetitive DNA sequences found in chromosomes. As a result, this methodology plays a role in exploring the genetic relationships among various plant groups [64,65]. Using fluorescence in situ hybridization mapping in diploid *A. morio*, six 18S-5.8S-25S rDNA sites and two 5S rDNA sites, or four 18S-5.8S-25S rDNA sites and two 5S rDNA sites in different populations, were identified [35,66]. The 18S-5.8S-25S rDNA sequence serves as a marker for nucleolus organising regions (NOR) and can be identified in chromosomes using the Ag-NOR technique. On the other hand, the 5S rDNA is a highly conserved coding sequence of 120 bp separated by an untranscribed variable spacer, which is separate from the 45S rDNA [67,68]. Variation in the number of 5S rDNA and 18S-5.8S-25S rDNA sites has been observed in other plant species, as reported in studies by Rogers et al. [69] and Panzera et al. [70]. On the other hand, only a few data points have been obtained for the genera *Himantoglossum*, *Ophrys*, and *Serapias*. In *S. vomeracea*, FISH revealed three pairs of 18S-5.8S-25S rDNA sites and two pairs of chromosomes with 5S rDNA sequences. In *H. hircinum*, in-situ hybridization showed four 18S-5.8S-25S rDNA sites and four 5S rDNA sites. Double-stained in situ hybridization of *H. robertianum* chromosomes revealed a pair with both pTa794 and pTa71 signals on opposite arms. In *O. tenthredinifera*, hybridization revealed two pairs of 5S rDNA and two pairs of 18S-5.8S-25S rDNA sites [35,66].

Furthermore, the data revealed variations in the number, location, and size of ribosomal sites, particularly in relation to 5S rDNA. Within the *Anacamptis* s.l. group, *A. collina* and *A. morio* had two 5S rDNA sites, while *A*. *papilionacea* had four or five. However, *Himantoglossum hircinum*, *Ophrys tenthredinifera*, and *Serapias vomeracea* exhibited four 5S rDNA sites. Interestingly, *Himantoglossum robertianum* displayed a pair of chromosomes carrying both 5S and 18S-25S signals on opposite arms [66]. The variability of 18S-25S rDNA sites was observed in all species examined ([35,66], this work), as reported in previous studies on other groups of *Orchidaceae* [71]. In contrast, Querino et al. [72] found stability in the number of 18S-25S rDNA sites in Laeliinae Benth. This study contributes to our understanding of the molecular cytogenetics of *Orchidinae* species and emphasises the need for further research in this area.

## 4. Materials and Methods

### 4.1. Cytological Analysis

Mitotic chromosomes were observed in the tissues of immature ovaries. At least ten metaphases were examined, and the karyotype was constructed from well-spread metaphase plates. Immature ovary tissues were pre-treated with 0.3% colchicine at room temperature for 2 h. For Feulgen staining, they were fixed for 5 min in 5:1:1:1 (*v/v*) absolute ethanol, chloroform, glacial acetic acid, and formalin. Hydrolysis was performed at 20 °C in 5.5 N HCl for 20 min [73]. The material was then stained with freshly prepared Feulgen stain.

For C-banding, immature ovaries were fixed in 3:1 (*v/v*) ethanol-glacial acetic acid and stored in the deep freezer for up to several months. Subsequently, they were squashed in 45% acetic acid; coverslips were removed by the dry ice method, and the preparations were air-dried overnight. Slides were then immersed in 0.2 N HCl at 60 °C for 3 min, thoroughly rinsed in distilled water, and then treated with 4% Ba(OH)2 at 20 °C for 4 min. After thorough rinsing, they were incubated in 2× SSC at 60 °C for 1 h. The stain used was 3–4% Giemsa (BDH) at pH 7.

For DAPI (4–6-diamidino-2-phenylindole) staining, ovaries were treated as for C-banding and stained using a buffered DAPI solution (0.6 mg/mL) for 5 min after which they were rinsed and mounted in 1:1 (*v/v*) buffer and glycerol. For chromomycin A3 (CMA) staining, slides were stained with 0.5 mg/mL CMA for 1 h and mounted in 1:1 (*v/v*) McIlvaine’s pH 7.0 buffer-glycerol. For identification of the nucleolus, AgNO3 precipitation was used [27].

Five well-spread metaphase plates were then examined with the FISH technique. For fluorescence in situ hybridization, the ribosomal sequences 18S-5.8S-25S (pTa71—red signals) and 5S (pTa794—green signals) were used as probes. Clone pTa71 was labelled with rhodamine-4-dUTP by nick translation, while pTa794 was labelled with digoxigenin-11-dUTP using a polymerase chain reaction. The former contains a 9kb *EcoBl* repeat unit of 18S-5.8S-25S rDNA and intergenic spacer regions, isolated from *Triticum aestivum* L. [74], and the latter corresponds to a complete 410 bp 5S gene unit, containing the 5S gene and intergenic spacer regions, isolated from *Triticum aestivum* [75]. The pre-treatment of slides and the FISH procedure followed the protocol in Heslop-Harrison [76]. The chromosomes and DNA probes were denatured together at 70 °C for 5 min and hybridization was performed at 37 °C overnight. After hybridization, the coverslips were removed in 2× SSC at room temperature and then washed thoroughly for 10 min in 20% (*v/v*) formamide in 0.1× SSC at 42 °C to remove sequences with less than 85% homology; the slides were then incubated in immunofluorescent reagents. For detection of the digoxigenin-labelled probe, the slides were equilibrated in 4× SSC/0.1% (*v/v*) Tween 20 and blocked in 5% (*w*/*v*) bovine serum albumin in 4× SSC/0.1% (*v/v*) Tween 20 for 5 min. Slides were incubated with sheep anti-digoxigenin antibody conjugated with FITC in a moist chamber at 37 °C for 1 h. The slides were washed in 4× SSC/Tween 20 for 3 × 5 min and subsequently counterstained with DAPI prior to observation. They were finally mounted in antifade solution AF1 (Citifluor) and examined with a Leitz epifluorescence microscope with single and triple band-pass filters.

The resulting images were processed with free image-editing software, applying the functions to the whole image.

### 4.2. Nomenclature

Regarding the nomenclature of species, we followed Delforge [15] and, in some cases, POWO [77].

### 4.3. Chromosome Numbers and Karyotype Parameters

Chromosome pairs were identified and arranged on the basis of length. The nomenclature used for describing karyotype composition follows Levan et al. [78], who distinguish centromeric position using the terms “median (arm ratio 1.0–1.7),” “submedian (a.r. 1.7–3.0),” “subterminal (a.r. 3.0–7.0),” and “terminal (a.r. 7.0-∞).” Karyotype morphometric characters were evaluated by calculating haploid complement length together with A1 (intrachromosomal asymmetry index), which measures the average position of the centromere in a karyotype, and A2 (interchromosomal asymmetry index), which measures variation in chromosome lengths [79]. Moreover, the karyotype asymmetry indices MCA (Mean Centromeric Asymmetry) and CVCL (Coefficient of Variation of Chromosome Length) were used for the evaluation of karyotype asymmetry [80,81,82].

Chromosome measurements were conducted using the freeware IdeoKar (http://agri.uok.ac.ir/ideokar/index.html, accessed on 10 May 2023).

Diagrams of the A1/A2 values and those of Mca/CVcl of the karyotypes were generated through the OpenOffice 4.1.14 program.

## 5. Conclusions

Cytogenetic analysis plays a crucial role in understanding evolutionary pathways and aiding in phylogenetic reconstruction. This method has been extensively used in studies focused on the phylogeny of orchids, and it has significantly influenced the classification of the *Orchidinae* subtribe. Researchers have analysed various entities, including species, subspecies, and hybrids, that belong to the *Orchidinae* subtribe. They have studied different karyological parameters such as chromosome number, karyotype morphology, distribution, and composition of constitutive heterochromatin. Additionally, fluorescent in situ hybridization (FISH) has been utilised in some species to locate the genes of the 18S-5.8S-28S rDNA and 5S rDNA ribosomal complexes on the chromosomes.

While traditional cytogenetic techniques have proven useful in studying Orchidaceae, integrating additional molecular cytogenetic methods is essential for future research in this field. By localising specific DNA sequences on chromosomes and identifying individual chromosomes in a karyotype, researchers can gain a more comprehensive understanding of taxonomic characteristics. One such method is fluorescence in situ hybridization, which has been highly effective in studying several orchid genera. In the *Anacamptis* genus, this technique has been employed to study *A. collina*, *A. morio*, *A. papilionacea*, and the interspecific hybrid *A.* × *gennarii*. Similarly, this approach has been applied to the *Himantoglossum* genus, examining *H. hircinum* and *H. robertianum*, as well as the *Serapias* genus with *S. vomeracea* and the species *O. tenthredinifera* in the genus *Ophrys*.

In the subtribe *Orchidinae*, many species have a diploid chromosome number of 2n = 32, 36, 40, and 42. However, polyploidy can also occur, resulting in chromosomal numbers such as 2n = 54, 63, 72, 80, and 84. Another source of variation is the presence of supernumerary chromosomes, known as B chromosomes, which have been observed in orchid species like *Anacamptis coriophora*, *A. palustris*, *A. papilionacea*, *Dactylorhiza romana* (Sebast.) Soó, *D. urvilleana* (Steudel) H.Baumann and Künkele, *Neotinea lactea* (Poir.) R.M.Bateman, Pridgeon and M.W.Chase, *N. tridentata* (Scop.) R.M.Bateman, Pridgeon and M.W.Chase, *N. ustulata* (L.) R.M.Bateman, Pridgeon and M.W.Chase, *Ophrys bertolonii*, *O. scolopax*, and *Orchis mascula* (L.) L. Cytological and molecular studies suggest that most of these B chromosomes arise from the autosomal complement of their host species [83,84,85]. Moreover, cellular mechanisms can cause heterochromatinization of these extra elements, leading to the differentiation of the supernumerary chromosome [86]. The Giemsa banding method has been used to identify heterochromatic supernumerary chromosomes in *Dactylorhiza romana* and *D. urvilleana*, with the accessory chromosomes observed in these species being similar in size to the longer chromosomes present in the complement [86,87].

Meiotic analysis has also provided valuable insights into the genetic makeup of some polyploid species and diploid/triploid hybrids in the *Orchidinae* subtribe. In particular, the observation of trivalent figures during Metaphase I of meiosis in triploid species such as *Anacamptis laxiflora* and *A. pyramidalis* confirms their autotriploid origin.

- The study of epigenetic effects in orchid species is an intriguing and relatively new field of research, although only a few documented cases have been reported thus far. Epigenetics focuses on investigating heritable changes in phenotype that occur without altering the DNA sequence [88,89]. Some researchers have observed transcriptional activity in specific orchid species, suggesting a potential role for epigenetic factors. In certain *Dactylorhiza* species with variations in geographic and ecological contexts, it has been demonstrated that ecological divergence is primarily influenced by epigenetic factors that regulate gene expression in response to environmental stimuli [90]. Based on these fascinating discoveries and considering the intricate morphological, cytogenetic, and molecular complexities within *Ophrys* species, it is conceivable that similar epigenetic processes occur across numerous entities within this group. Such processes could potentially account for the formation of various microspecies and the notable phenotypic variation observed [91].

In conclusion, the study of orchid cytogenetics has uncovered a remarkable level of complexity and variability within this plant group. Classical cytogenetic techniques have proven invaluable in elucidating taxonomic relationships, characterising individual chromosomes, and unravelling the mechanisms underlying polyploidy and supernumerary chromosomes. Meiotic analysis has provided important insights into the origin of hybrids and polyploid species. Furthermore, the investigation of epigenetic effects holds promise for understanding the adaptation and evolution of orchids, particularly in relation to their ecological context and interactions with pollinators. The integration of classical and molecular cytogenetic approaches, along with meiotic and epigenetic analyses, will continue to shed light on the intricate genetic and genomic aspects of orchids, advancing our understanding of their diversity, evolution, and ecological significance.

## Figures and Tables

**Table 1:** Taxon, code, sample’s provenance, formula, and morphometric parameters in *Anacamptis*, *Serapias* and *Himantoglossum* (average values). THL = Total chromosome length of the haploid complement; M_CA_ = Mean Centromeric Asymmetry; CV_CL_ = Coefficient of Variation of Chromosome Length; CV_CI_ = Coefficient of Variation of Centromeric Index; and Intrachromosomal (A1) and interchromosomal (A2) asymmetry indices. Chromosome abbreviations: *m*, metacentric; *sm*, submetacentric; *st*, subtelocentric.

Taxon	Code	Provenance	Formula	THL	M_CA_	CV_CL_	CV_CI_	A1	A2
*Anacamptis morio* (L.) R.M.Bateman, Pridgeon & M.W.Chase	mor	Italy	32 *m* + 4 *sm*	43.69	11.02	20.86	9.70	0.19	0.21
*A. papilionacea* (L.) R.M.Bateman, Pridgeon & M.W.Chase	pap		16 *m* + 10 *sm* + 6 *st*	42.59	28.11	27.77	25.68	0.41	0.28
*A. pyramidalis* (L.) R.M.Bateman, Pridgeon & M.W.Chase 36	py36		30 *m* + 6 *sm*	51.58	16.27	31.25	12.24	0.26	0.31
*A. pyramidalis* 72	py72		44 *m* + 28 *sm*	94.64	22.52	35.43	19.52	0.34	0.35
*A. collina* (Banks & Sol. ex Russell) R.M.Bateman, Pridgeon & M.W.Chase	col		22 *m* + 14 *sm*	50.14	21.49	22.90	20.14	0.33	0.23
*A. coriophora* (L.) R.M.Bateman, Pridgeon & M.W.Chase	cor	Italy-Spain	16 *m* + 20 *sm*	57.50	24.58	21.49	16.72	0.38	0.21
*A. laxiflora* (Lam.) R.M.Bateman, Pridgeon & M.W.Chase	lax	Italy	32 *m* + 4*sm*	37.68	12.00	26.30	8.30	0.21	0.26
*A. palustris* (Jacq.) R.M.Bateman, Pridgeon & M.W.Chase	pal		30 *m* + 6*sm*	45.95	19.46	19.81	12.35	0.32	0.2
*Serapias vomeracea* (Burm.f.) Briq.	svo		6 *m* + 18 *sm* + 12 *st*	41.79	39.71	26.49	29.48	0.5	0.26
*S. bergonii* E.G.Camus	sbe		8 *m* + 24 *sm* + 4 *st*	40.96	35.36	27.78	18.70	0.51	0.28
*S. orientalis* s.l.	sap		6 *m* + 22 *sm* + 8 *st*	39.89	40.53	26.22	24.51	0.54	0.26
*S. parviflora* Parl.	spa		16 *m* + 18 *sm* + 2 *st*	40.87	27.21	29.12	20.07	0.4	0.27
*S. cordigera* L.	sco		22 *m* + 14 *sm*	39.28	27.57	37.22	15.73	0.42	0.38
*S. lingua* L. 72	sli72		36 *m* + 34 *sm* + 2 *st*	63.34	27.64	27.63	17.56	0.42	0.28
*S. politisii* Renz	spo		22 *m* + 10 *sm* + 4 *st*	40.47	24.91	29.08	24.86	0.39	0.28
*Himantoglossum hircinum* (L.) Spreng.	hhi		32 *m* + 4 *sm*	46.06	13.00	25.53	8.66	0.22	0.25
*H. robertianum* (Loisel.) P.Delforge	hro		32 *m* + 4 *sm*	62.80	14.49	36.37	10.60	0.24	0.36
*H. adriaticum* H.Baumann	had		30 *m* + 6 *sm*	45.14	17.61	28.42	12.33	0.29	0.29

**Table 2:** Taxon, code, sample’s provenance, formula, and morphometric parameters in *Ophrys* (average values). THL = Total chromosome length of the haploid complement; M_CA_ = Mean Centromeric Asymmetry; CV_CL_ = Coefficient of Variation of Chromosome Length; CV_CI_ = Coefficient of Variation of Centromeric Index; and Intrachromosomal (A1) and interchromosomal (A2) asymmetry indices. Chromosome abbreviations: *m*, metacentric; *sm*, submetacentric.

Taxon	Code	Provenance	Formula	THL	M_CA_	CV_CL_	CV_CI_	A1	A2
*Ophrys apifera* Huds.	opi	Italy	24 *m* + 12 *sm*	48.18	17.82	27.72	15.99	0.3	0.28
*O. bertolonii* Moretti	obe		18 *m* + 18 *sm*	45.09	23.77	23.05	18.70	0.38	0.25
*O. bombyliflora* Link	obo		32 *m* + 4 *sm*	43.61	16.20	21.72	10.13	0.27	0.2
*O. ferrum-equinum* Desf.	ofe	Greece	18 *m* + 18 *sm*	41.60	21.52	23.96	14.35	0.38	0.26
*O. fusca* s.l.	ofu	Italy	32 *m* + 4 *sm*	40.25	14.83	21.99	14.38	0.27	0.22
*O. iricolor* Desf.	oir		22 *m* + 14 *sm*	43.85	19.75	23.71	15.53	0.32	0.23
*O. pallida* Raf.	opl		26 *m* + 10 *sm*	44.79	15.67	21.27	9.24	0.33	0.22
*O. apulica* (O.Danesch & E.Danesch) O.Danesch & E.Danesch	oap		20 *m* + 16 *sm*	46.86	21.08	28.46	15.67	0.33	0.28
*O. lacaitae* Lojac.	ola		20 *m* + 16 *sm*	47.43	24.42	26.86	20.75	0.39	0.27
*O. celiensis* (O.Danesch & E.Danesch) P.Delforge	oox		20 *m* + 16 *sm*	44.10	23.19	23.14	20.64	0.36	0.23
*O. parvimaculata* (O.Danesch & E.Danesch) Paulus & Gack	opa		18 *m* + 18 *sm*	40.04	22.79	28.42	21.07	0.4	0.29
*O. insectifera* L.	oin		10 *m* + 26 *sm*	50.74	28.00	25.25	22.01	0.43	0.25
*O. lutea* Cav.	olu		28 *m* + 8 *sm*	43.41	18.72	22.72	12.09	0.3	0.23
*O. sicula* Tineo	osi		28 *m* + 8 *sm*	45.40	18.28	21.46	9.47	0.3	0.21
*O. conradiae* Melki & Deschatres	oco		22 *m* + 14 *sm*	51.34	21.09	20.98	17.32	0.33	0.21
*O. classica* Devillers-Tersch. & Devillers	osp		26 *m* + 10 *sm*	47.47	20.93	23.39	13.89	0.35	0.24
*O. incubacea* Bianca	oat		26 *m* + 10 *sm*	42.91	19.55	22.24	12.42	0.32	0.22
*O. garganica* E.Nelson ex O.Danesch & E.Danesch	ops		24 *m* + 12 *sm*	44.87	20.36	24.47	15.52	0.33	0.25
*O. tenthredinifera* s.l.	ote		30 *m* + 6 *sm*	50.96	14.95	18.94	10.58	0.25	0.19
*O. umbilicate* Desf.	oum	Turkey	18 *m* + 18 *sm*	51.23	20.51	27.47	20.38	0.36	0.32
*O. biscutella* O.Danesch & E.Danesch	obi	Italy	20 *m* + 16 *sm*	45.45	20.48	26.05	16.21	0.35	0.26
*O. arachnitiformis* Gren. & Phil. (incl. *O. mateolana* Medagli, D’Emerico, Bianco & Ruggiero and *O. archipelagi* Gölz & H.R.Reinhard)	oar		20 *m* + 16 *sm*	50.07	22.08	25.37	20.99	0.36	0.26
*O. tarentina* Gölz & H.R.Reinhard	ota		26 *m* + 10 *sm*	44.95	21.26	22.78	16.19	0.31	0.23
*O. tardans* O.Danesch & E.Danesch	otr		32 *m* + 4 *sm*	41.64	16.72	20.26	10.17	0.25	0.2
*O. speculum* Link	ose	Turkey [15]	32 *m* + 4 *sm*	34.28	11.99	19.10	10.11	0.2	0.19
*O. omegaifera* H.Fleischm.	oom	[15]	28 *m* + 8 *sm*	39.90	15.34	19.52	11.01	0.25	0.21
*O. mammosa* Desf.	omm	[15]	22 *m* + 14 *sm*	33.34	20.76	21.06	16.78	0.32	0.21
*O. lycia* Renz & Taubenheim	oly	[15]	26 *m* + 10 *sm*	43.64	16.14	17.82	11.97	0.27	0.18
*O. reinholdii* Spruner ex H.Fleischm.	ore	[15]	24 *m* + 12 *sm*	37.65	16.47	21.69	13.63	0.3	0.2

## Data Availability

Data are contained in the article.
